# Crystal structures of two new 3-(2-chloro­eth­yl)-*r*(2),*c*(6)-diarylpiperidin-4-ones

**DOI:** 10.1107/S2056989018003766

**Published:** 2018-03-09

**Authors:** K. Rajkumar, S. Sivakumar, R. Arulraj, Manpreet Kaur, Jerry P. Jasinski, A. Manimekalai, A. Thiruvalluvar

**Affiliations:** a Research and Development Centre, Bharathiar University, Coimbatore 641 046, Tamilnadu, India; b Department of Chemistry, Thiruvalluvar Arts and Science College, Kurinjipadi 607 302, Tamilnadu, India; c Department of Chemistry, Keene State College, 229 Main Street, Keene, NH, 03435-2001, USA; d Department of Chemistry, Annamalai University, Annamalai Nagar 608 002, Tamilnadu, India; e Principal, Kunthavai Naacchiyaar Government Arts College for Women (Autonomous), Thanjavur 613 007, Tamilnadu, India

**Keywords:** crystal structure, bifurcated bond, weak inter­molecular hydrogen bonds

## Abstract

The syntheses and crystal structures of 3-(2-chloro­eth­yl)-*r*-2,*c*-6-di­phenyl­piperidin-4-one, C_19_H_20_ClNO, (I), and 3-(2-chloro­eth­yl)-*r*-2,*c*-6-bis­(4-fluoro­phen­yl)piperidin-4-one, C_19_H_18_ClF_2_NO, (II), are described. The piperidone ring adopts a chair conformation in (I), whereas a slightly distorted chair conformation is formed in (II). The dihedral angle between the mean plane of the phenyl rings is 59.1 (1)° in (I) and 76.1 (1)° in (II). The crystal packing features weak inter­molecular N—H⋯O hydrogen bonds in each structure.

## Abstract 2

The syntheses and crystal structures of 3-(2-chloro­eth­yl)-*r*-2,*c*-6-di­phenyl­piperidin-4-one C_19_H_20_ClNO and 3-(2-chloro­eth­yl)-*r*-2,*c*-6- bis­(*p*-fluoro­phen­yl)piperidin-4-one C_19_H_18_ClF_2_NO are described.

## Chemical context   

Piperidone mol­ecules exhibit a wide spectrum of biological activities ranging from anti-bacterial to anti-cancer (Parthiban *et al.*, 2005 ▸, 2009 ▸, 2011 ▸). Most of the 2,6-diaryl-substituted piperidones and their derivatives are of significant pharmacological importance (Aridoss *et al.*, 2007 ▸). Some novel 3,5-di­chloro-2,6-di­aryl­piperidin-4-ones are also reported to possess anti­microbial activity (Bhakiaraj *et al.*, 2014 ▸). Piperidones also display analgesic, anti-inflammatory, central nervous system (CNS), local anaesthetic, anti­cancer and anti­microbial activity (Perumal *et al.*, 2001 ▸). In view of the relevance of piperidone derivatives to a variety of ongoing health and pharmalogical issues, we have synthesized the title compounds and report their crystal structures here. Arulraj *et al.* (2017 ▸) has reported the crystal structure of three related 3-chloro-3-methyl-2,6-di­aryl­piperidin-4-ones. In each of these structures, the piperidine rings adopt chair conformations similar to what we have observed in the title compounds.
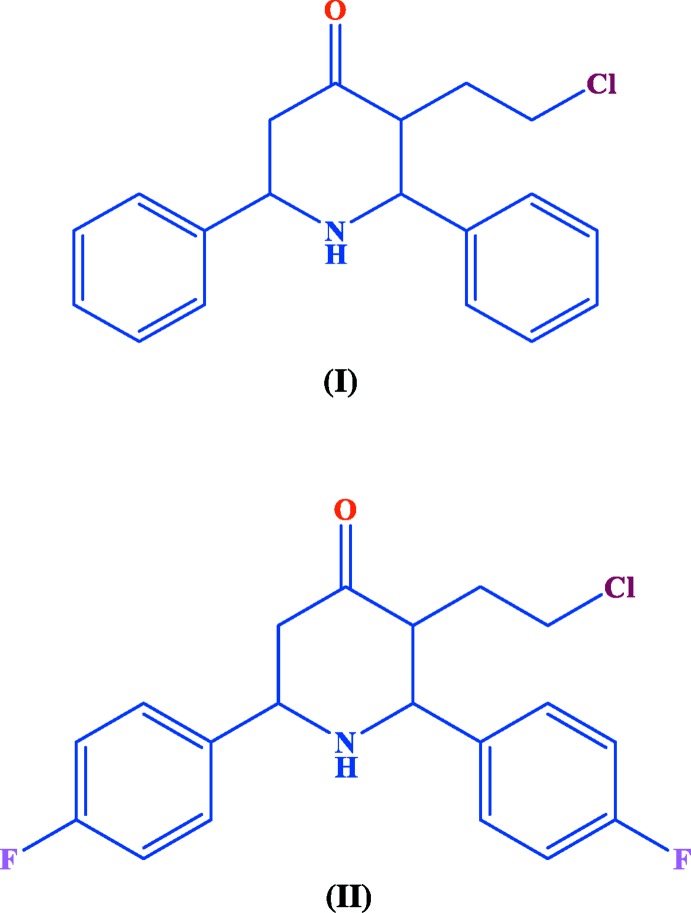


## Structural commentary   

**Figure 1:**
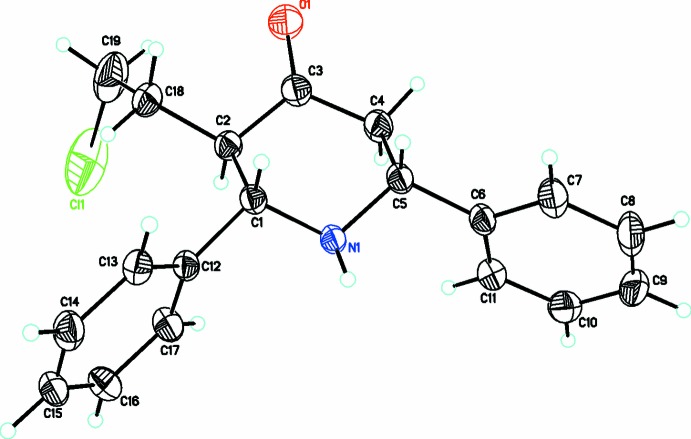
A view of the mol­ecular structure of (I), showing displacement ellipsoids drawn at the 30% probability level.

**Figure 2:**
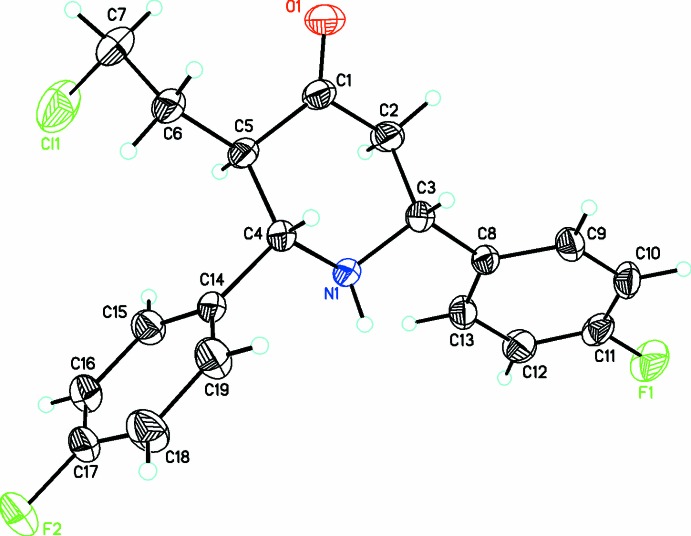
A view of the mol­ecular structure of (II), showing displacement ellipsoids drawn at the 30% probability level.

Two new 3-(2-chloro­eth­yl)-*r*-2,*c*-6-di­aryl­piperidin-4-one compounds, C_19_H_20_ClNO (I) and C_19_H_18_ClF_2_NO (II), each crystallize in the *P*2_1_/*c* space group with one independent mol­ecule in the asymmetric unit. The piperidone ring adopts a chair conformation in (I), (Fig. 1 ▸), whereas it forms a slightly distorted chair conformation in (II), (Fig. 2 ▸), with puckering parameters *Q* = 0.576 (2) Å, θ = 164.2 (2)°, φ = 179.4 (8)° in (I) and *Q* = 0.601 (2) Å, θ = 4.93 (19)°, φ = 356 (2)° in (II). The dihedral angle between the mean planes of the phenyl rings is 59.1 (1)° in (I) and 76.1 (1)° in (II). The increase in this dihedral angle in (II) could be attributed to steric repulsion from the substituent fluorine atoms. The sum of the bond angles around N1 in each structure [332.5° in (I) and 331.9° in (II)] is consistent with sp^3^ hybridization (Beddoes *et al.*, 1986 ▸).

The substituents on the piperidine ring in both (I) and (II) adopt equatorial orientations with the keto oxygen atom being anti-clinal [O1—C3—C4—C5 = 136.1 (2)°] in (I) and anti-periplanar [O1—C1—C5—C4 = −120.4 (2)°] in (II). The 2-chloro­ethyl group lies in a syn-clinal orientation in both (I) [C3—C2—C18—C19 = 75.6 (3)°] and (II) [C1—C5—C6—C7 = 76.4 (2)°]. The two diaryl groups are both anti-clinal [N1—C5—C6—C11 = 54.5 (3)° and N1—C1—C12—C13 = 123.97 (18)°] in (I) whereas in (II) they are both syn-clinal [N1—C4—C14—C15 = −78.4 (2)° and N1—C3—C8—C13 = 35.4 (2)°].

## Supra­molecular features   

**Figure 3:**
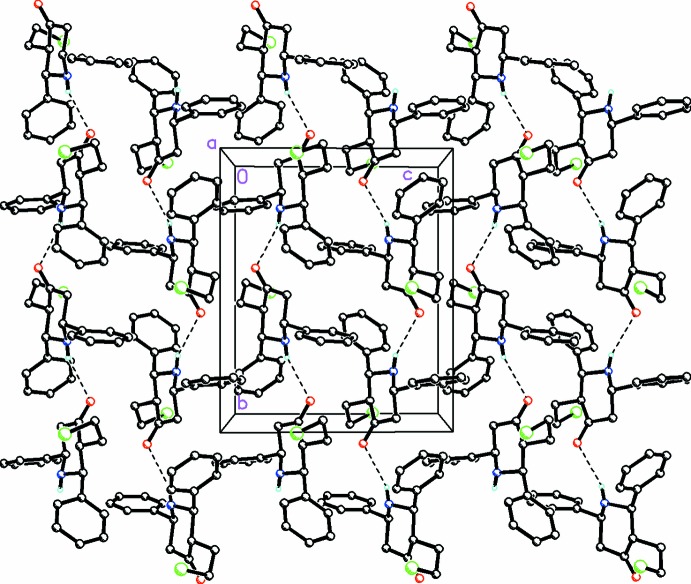
A partial view along the *a* axis of the crystal packing for (I), showing infinite chains formed along [010] by weak N1—H1⋯O1 hydrogen bonds with the mol­ecules rotating in a 180° spiral motif along the axis. H atoms not involved in this inter­action have been omitted for clarity.

The crystal packing features very weak N1—H1⋯O1 hydrogen bonds in (I), forming infinite *C*(6) chains along the *b-*axis direction, with the mol­ecules rotating in a 180° spiral motif along the axis (Table 1 ▸, Fig. 3 ▸). In addition, a weak C—H⋯π inter­action between the piperdine ring and a diaryl group in (I) also occurs.

**Figure 4:**
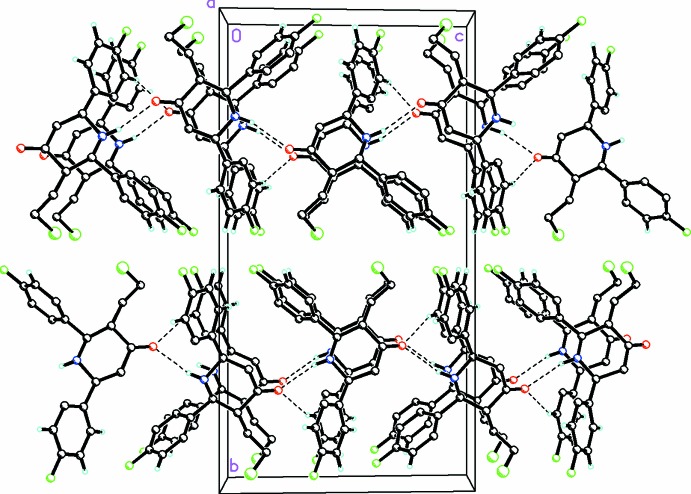
A partial view along the *a* axis of the crystal packing for (II), showing infinite chains formed along [001] by weak N1—H1⋯O1 and C12—H12⋯O1 hydrogen-bonding inter­actions. The keto oxygen, O1, forms a weak hydrogen bond with N1 from a piperdine ring in the same plane and with C12 from one of the diaryl groups of a mol­ecule in an adjacent plane along the *a* axis. H atoms not involved in these inter­actions have been omitted for clarity.

In (II), weak N—H⋯O hydrogen bonds (Fig. 4 ▸, Table 2 ▸) are again observed, also forming infinite *C*(6) chains but along the *c* axis in this case. Weak C—H⋯O and C—H⋯F inter­actions (Table 2 ▸) are also observed and contribute to the packing stability. In (II), the keto oxygen, O1, acts as the acceptor of weak hydrogen bonds involving atom N1 from a piperdine ring in the same plane and with atom C12 from one of the diaryl groups of a mol­ecule in an adjacent plane along the *a* axis. An unusual weak C1—O1⋯π [O1⋯π = 3.8263 (19) Å, C1⋯π = 4.377 (2) Å, C1—O1⋯π = 109°; *x*, 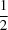 − *y*, −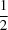 + *z*; centroid of the C8–C13 ring] inter­action also between the piperidine ring and a diaryl group is observed.

## Database survey   

A search in the Cambridge Crystallographic Database (CSD version 5.38 of Nov, 2016, updates May, 2017; Groom *et al.*, 2016 ▸) for the 2,6-di­phenyl­piperidin-4-one skeleton resulted in 229 hits, which was further refined to 50 hits by removing those structures in which the title skeleton substructure was combined with larger mol­ecules. The two most closely related remaining structures based on the pendant arms of the 2,6-di­phenyl­piperidine-4-one central substructure, *viz*. 2,6-diphenyl-3-iso­propyl­piperidin-4-one (ACEZUD; Nilofar Nissa *et al.*, 2001 ▸) and *t*-3-pentyl-r-2,c-6-di­phenyl­piperidin-4-one (RUGLOV; Gayathri *et al.*, 2009 ▸) were then compared with the two reported here. The piperidone ring in compounds (I) and (II) reported here adopt chair or distorted chair conformations, unlike in ACEZUD and RUGLOV. The crystal packing is stabilized by N—H⋯O inter­molecular hydrogen bonds in both (I) and (II), as well as in ACEZUD. In contrast, the crystal packing in RUGLOV is influenced only by weak C—H⋯O and C—H⋯π inter­molecular inter­actions.

## Synthesis and crystallization   

A mixture of ammonium acetate (0.1 mol, 7.71 g), the respective aldehyde (0.2 mol), benzaldehyde/*p*-fluoro­benzaldehyde (20.4 ml/21.0 ml) and 5-chloro-2-penta­none (0.1 mol, 11.4 ml) in distilled ethanol was heated first to boiling. After cooling, the viscous liquid obtained was dissolved in diethyl ether (200 ml) and shaken with 100 ml of concentrated hydro­chloric acid. The precipitated hydro­chlorides of the 3-(2-chloro­eth­yl)-*r*-2,*c*-6-di­aryl­piperidin-4-ones were removed by filtration and washed first with a 40 ml mixture of ethanol and diethyl ether (1:1) and then with diethyl ether to remove most of the coloured impurities. The base was liberated from an alcoholic solution by adding aqueous ammonia and then diluted with water. Each compound was recrystallized twice from a distilled ethanol solution: single crystals of (I) and (II) were obtained after two days. The yield of the isolated product was 3.0 g (I) and 2.5 g (II).

**3-(2-Chloro­eth­yl)-*r*-2,*c*-6-di­phenyl­piperidin-4-one, (C_19_H_20_ClNO), (I):**

IR (KBr): 3311.07 (νN—H), 3067.56, 3033.34 (νC—H), 1697.03 (νC=O), 1605.39, 1493.90 (νC=C), 769.33 (νC—Cl) cm^−1. 1^H NMR (400 MHz, CDCl_3_): δ 7.42–7.19 (*m*, aromatic protons), 4.03 (*d*, H6 proton), 3.64 (*d*, H2 proton), 3.36–3.33 (m, H5a proton), 2.61 (*dd*, H5e proton), 2.18–2.09 (*m*, H3 proton, 1.99 (*s*, NH proton), 2.94 (*s*, CH_2_Cl proton), 2.75 (*t*, CH_2_ proton). ^13^C NMR (CDCl_3_, 400 MHz): δ 208.60 (C=O), 140.67 (aromatic *ipso* carbon atoms), 128.81–126.63 (aromatic carbon atoms), 67.27 (C-3 carbon), 61.92 (C-2 carbon), 53.76 (C-6 carbon), 51.27 (C-5 carbon), 28.18 (methyl­ene carbon), 43.49 (CH_2_Cl Carbon). Melting point: 371 K.

**3-(2-Chloro­eth­yl)-** ***r*** **-2**,***c*** **-6-bis­(p-fluoro­phen­yl)piperidin-4-one, (C_19_H_18_ClF_2_NO), (II):**

IR (KBr): 3292.53 (νN—H), 3078.27, 3077.86 (νC—H), 1702.32 (νC=O), 1605.79, 1511.47 (νC=C), 760.50 (νC—Cl) cm^−1. 1^H NMR (400 MHz, CDCl_3_): δ 7.39–7.02 (*m*, aromatic protons), 3.99 (*dd*, H6 proton), 3.61 (*d*, H2 proton), 3.36 (*dd*, H5a proton), 2.52 (*dd*, H5e proton), 2.16–2.08 (*m*, H3 proton), 1.99 (*s*, NH proton), 2.84 (*t*, CH_2_Cl proton), 2.67 (*t*, CH_2_ proton). ^13^C NMR (CDCl_3_, 400 MHz): δ 208.09 (C=O), (aromatic *ipso* carbon atoms), 115.84–115.51 (aromatic carbon atoms), 55.77 (C-3 carbon), 66.34 (C-2 carbon), 61.09 (C-6 carbon), 51.41 (C-5 carbon), 28.06 (methyl­ene carbon), 43.45 (CH_2_Cl Carbon). Melting point: 375 K.

## Refinement   

Crystal data, data collection and structure refinement details are summarized in Table 3 ▸. The N1-bound H atoms in both mol­ecules were located in a difference-Fourier map and their coordinates and displacement parameters freely refined. All C-bound H atoms were refined using a riding model with *d*(C—H) = 0.93 Å for aromatic, 0.97 Å for methyl­ene and 0.98 Å for methine H atoms, all with *U*_iso_ = 1.2*U*_eq_ (C)

## Supplementary Material

Crystal structure: contains datablock(s) I, II, global. DOI: 10.1107/S2056989018003766/sj5546sup1.cif

Structure factors: contains datablock(s) I. DOI: 10.1107/S2056989018003766/sj5546Isup2.hkl

Structure factors: contains datablock(s) II. DOI: 10.1107/S2056989018003766/sj5546IIsup3.hkl

Supporting information file. DOI: 10.1107/S2056989018003766/sj5546Isup4.cml

Supporting information file. DOI: 10.1107/S2056989018003766/sj5546IIsup5.cml

CCDC references: 1827383, 1827382

Additional supporting information:  crystallographic information; 3D view; checkCIF report

## Figures and Tables

**Table 1:** Hydrogen-bond geometry (Å, °) for (I) *Cg*3 is the centroid of the C12–C17 ring.

*D*—H⋯*A*	*D*—H	H⋯*A*	*D*⋯*A*	*D*—H⋯*A*
N1—H1⋯O1^i^	0.86 (2)	2.52 (2)	3.335 (2)	158 (2)
C4—H4*A*⋯*Cg*3^ii^	0.97	2.79	3.665 (2)	150

Symmetry codes: (i) 
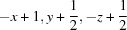
; (ii) 
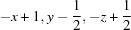
.

**Table 2:** Hydrogen-bond geometry (Å, °) for (II)

*D*—H⋯*A*	*D*—H	H⋯*A*	*D*⋯*A*	*D*—H⋯*A*
N1—H1⋯O1^i^	0.89 (2)	2.32 (2)	3.189 (2)	165 (2)
C9—H9⋯F2^ii^	0.93	2.61	3.378 (2)	140
C10—H10⋯F2^iii^	0.93	2.58	3.343 (2)	139
C12—H12⋯O1^iv^	0.93	2.57	3.412 (3)	150
C16—H16⋯F1^v^	0.93	2.62	3.379 (2)	139

Symmetry codes: (i) 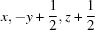; (ii) 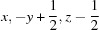; (iii) 
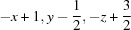
; (iv) 
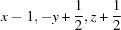
; (v) 
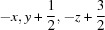
.

**Table 3:** Experimental details

	(I)	(II)
Crystal data		
Chemical formula	C_19_H_20_ClNO	C_19_H_18_ClF_2_NO
*M*_r_	313.81	349.79
Crystal system, space group	Monoclinic, *P*2_1_/*c*	Monoclinic, *P*2_1_/*c*
Temperature (K)	293	293
*a*, *b*, *c* (Å)	11.3306 (3), 13.3638 (4), 10.9821 (3)	5.5105 (2), 24.2612 (6), 12.8622 (3)
β (°)	91.996 (2)	93.809 (3)
*V* (Å^3^)	1661.90 (8)	1715.77 (9)
*Z*	4	4
Radiation type	Cu *K*α	Cu *K*α
μ (mm^−1^)	2.03	2.20
Crystal size (mm)	0.42 × 0.38 × 0.14	0.34 × 0.16 × 0.14

Data collection		
Diffractometer	Rigaku Oxford Diffraction	Rigaku Oxford Diffraction
Absorption correction	Multi-scan (*CrysAlis PRO*; Rigaku OD, 2015 ▸)	Multi-scan (*CrysAlis PRO*; Rigaku OD, 2015 ▸)
*T*_min_, *T*_max_	0.535, 1.000	0.524, 1.000
No. of measured, independent and observed [*I* > 2σ(*I*)] reflections	6237, 3168, 2545	6548, 3267, 2702
*R*_int_	0.028	0.020
(sin θ/λ)_max_ (Å^−1^)	0.615	0.614

Refinement		
*R*[*F* ^2^ > 2σ(*F* ^2^)], *wR*(*F* ^2^), *S*	0.055, 0.158, 1.05	0.046, 0.131, 1.04
No. of reflections	3168	3267
No. of parameters	204	222
H-atom treatment	H atoms treated by a mixture of independent and constrained refinement	H atoms treated by a mixture of independent and constrained refinement
Δρ_max_, Δρ_min_ (e Å^−3^)	0.56, −0.44	0.34, −0.38

Computer programs: *CrysAlis PRO* (Rigaku OD, 2015 ▸), *SHELXT* (Sheldrick, 2015*a* ▸), *SHELXL2018* (Sheldrick, 2015*b* ▸) and *OLEX2* (Dolomanov *et al.*, 2009 ▸).

## supplementary crystallographic information

### 3-(2-Chloroethyl)-r-2,c-6-diphenylpiperidin-4-one (I) Crystal data

**Table:**

C_19_H_20_ClNO	*F*(000) = 664
*M**_r_* = 313.81	*D*_x_ = 1.254 Mg m^−^^3^
Monoclinic, *P*2_1_/*c*	Cu *K*α radiation, λ = 1.54184 Å
*a* = 11.3306 (3) Å	Cell parameters from 2185 reflections
*b* = 13.3638 (4) Å	θ = 5.1–71.2°
*c* = 10.9821 (3) Å	µ = 2.03 mm^−^^1^
β = 91.996 (2)°	*T* = 293 K
*V* = 1661.90 (8) Å^3^	Prism, colourless
*Z* = 4	0.42 × 0.38 × 0.14 mm

### 3-(2-Chloroethyl)-r-2,c-6-diphenylpiperidin-4-one (I) Data collection

**Table:**

Rigaku Oxford Diffraction diffractometer	3168 independent reflections
Radiation source: fine-focus sealed X-ray tube, Enhance (Cu) X-ray Source	2545 reflections with *I* > 2σ(*I*)
Graphite monochromator	*R*_int_ = 0.028
Detector resolution: 16.0416 pixels mm^-1^	θ_max_ = 71.4°, θ_min_ = 3.9°
ω scans	*h* = −13→13
Absorption correction: multi-scan (CrysAlis PRO; Rigaku OD, 2015)	*k* = −14→16
*T*_min_ = 0.535, *T*_max_ = 1.000	*l* = −13→8
6237 measured reflections	

### 3-(2-Chloroethyl)-r-2,c-6-diphenylpiperidin-4-one (I) Refinement

**Table:**

Refinement on *F*^2^	Hydrogen site location: mixed
Least-squares matrix: full	H atoms treated by a mixture of independent and constrained refinement
*R*[*F*^2^ > 2σ(*F*^2^)] = 0.055	*w* = 1/[σ^2^(*F*_o_^2^) + (0.075*P*)^2^ + 0.5181*P*] where *P* = (*F*_o_^2^ + 2*F*_c_^2^)/3
*wR*(*F*^2^) = 0.158	(Δ/σ)_max_ < 0.001
*S* = 1.05	Δρ_max_ = 0.56 e Å^−^^3^
3168 reflections	Δρ_min_ = −0.44 e Å^−^^3^
204 parameters	Extinction correction: SHELXL2018 (Sheldrick, 2015b), Fc^*^=kFc[1+0.001xFc^2^λ^3^/sin(2θ)]^-1/4^
0 restraints	Extinction coefficient: 0.0026 (4)
Primary atom site location: dual	

### 3-(2-Chloroethyl)-r-2,c-6-diphenylpiperidin-4-one (I) Special details

**Table:**

Geometry. All esds (except the esd in the dihedral angle between two l.s. planes) are estimated using the full covariance matrix. The cell esds are taken into account individually in the estimation of esds in distances, angles and torsion angles; correlations between esds in cell parameters are only used when they are defined by crystal symmetry. An approximate (isotropic) treatment of cell esds is used for estimating esds involving l.s. planes.

### 3-(2-Chloroethyl)-r-2,c-6-diphenylpiperidin-4-one (I) Fractional atomic coordinates and isotropic or equivalent isotropic displacement parameters (Å^2^)

**Table:**

	*x*	*y*	*z*	*U*_iso_*/*U*_eq_	
Cl1	0.08089 (8)	0.50912 (12)	0.17386 (10)	0.1402 (6)	
O1	0.44751 (18)	0.41543 (13)	0.1217 (2)	0.0771 (6)	
N1	0.48661 (13)	0.69143 (12)	0.26049 (15)	0.0370 (4)	
H1	0.511 (2)	0.7523 (19)	0.270 (2)	0.045 (6)*	
C1	0.41112 (15)	0.67867 (13)	0.15013 (17)	0.0356 (4)	
H1A	0.460437	0.682251	0.078697	0.043*	
C2	0.35356 (16)	0.57351 (14)	0.15481 (18)	0.0393 (4)	
H2	0.303654	0.572734	0.225915	0.047*	
C3	0.44765 (19)	0.49404 (15)	0.1762 (2)	0.0479 (5)	
C4	0.5424 (2)	0.51707 (15)	0.2711 (2)	0.0509 (5)	
H4A	0.607257	0.470401	0.262937	0.061*	
H4B	0.511036	0.508140	0.351412	0.061*	
C5	0.58905 (17)	0.62451 (15)	0.25931 (18)	0.0410 (4)	
H5	0.627033	0.631318	0.180923	0.049*	
C6	0.67816 (17)	0.64909 (15)	0.3605 (2)	0.0447 (5)	
C7	0.79567 (19)	0.66364 (18)	0.3344 (2)	0.0557 (6)	
H7	0.819078	0.661112	0.254103	0.067*	
C8	0.8789 (2)	0.6821 (2)	0.4283 (3)	0.0711 (8)	
H8	0.957634	0.691906	0.410133	0.085*	
C9	0.8460 (2)	0.6858 (2)	0.5464 (3)	0.0744 (8)	
H9	0.902038	0.697968	0.608508	0.089*	
C10	0.7293 (3)	0.6715 (2)	0.5735 (3)	0.0688 (7)	
H10	0.706558	0.673952	0.654000	0.083*	
C11	0.6462 (2)	0.65346 (19)	0.4813 (2)	0.0575 (6)	
H11	0.567619	0.644106	0.500381	0.069*	
C12	0.31935 (15)	0.76066 (14)	0.14123 (16)	0.0361 (4)	
C13	0.30967 (17)	0.82110 (15)	0.03920 (18)	0.0420 (4)	
H13	0.362040	0.812853	−0.023373	0.050*	
C14	0.2224 (2)	0.89410 (17)	0.0292 (2)	0.0514 (5)	
H14	0.216607	0.933973	−0.040138	0.062*	
C15	0.14510 (19)	0.90767 (17)	0.1206 (2)	0.0544 (6)	
H15	0.086504	0.956250	0.113438	0.065*	
C16	0.1547 (2)	0.84889 (19)	0.2234 (2)	0.0574 (6)	
H16	0.102923	0.858368	0.286259	0.069*	
C17	0.24084 (19)	0.77582 (18)	0.23368 (19)	0.0488 (5)	
H17	0.246249	0.736357	0.303402	0.059*	
C18	0.2733 (2)	0.55077 (18)	0.0436 (2)	0.0510 (5)	
H18A	0.232158	0.611677	0.019567	0.061*	
H18B	0.322225	0.531314	−0.023166	0.061*	
C19	0.1841 (3)	0.4707 (3)	0.0620 (4)	0.0939 (11)	
H19A	0.223702	0.409828	0.088879	0.113*	
H19B	0.142107	0.456775	−0.014624	0.113*	

### 3-(2-Chloroethyl)-r-2,c-6-diphenylpiperidin-4-one (I) Atomic displacement parameters (Å^2^)

**Table:**

	*U*^11^	*U*^22^	*U*^33^	*U*^12^	*U*^13^	*U*^23^
Cl1	0.0686 (5)	0.2364 (16)	0.1155 (8)	−0.0747 (8)	−0.0010 (5)	0.0298 (8)
O1	0.0723 (12)	0.0455 (10)	0.1114 (15)	0.0094 (8)	−0.0271 (11)	−0.0258 (10)
N1	0.0310 (7)	0.0311 (8)	0.0486 (9)	0.0021 (6)	−0.0054 (6)	−0.0010 (6)
C1	0.0295 (8)	0.0354 (9)	0.0419 (9)	0.0005 (7)	0.0004 (7)	0.0006 (7)
C2	0.0343 (9)	0.0368 (10)	0.0466 (10)	−0.0024 (7)	−0.0015 (7)	−0.0015 (8)
C3	0.0436 (11)	0.0344 (10)	0.0654 (13)	−0.0010 (8)	−0.0037 (9)	−0.0017 (9)
C4	0.0487 (11)	0.0348 (10)	0.0681 (14)	0.0067 (9)	−0.0141 (10)	0.0006 (9)
C5	0.0335 (9)	0.0378 (10)	0.0514 (11)	0.0043 (8)	−0.0042 (8)	−0.0003 (8)
C6	0.0351 (9)	0.0359 (10)	0.0625 (12)	0.0061 (8)	−0.0092 (8)	−0.0008 (8)
C7	0.0397 (11)	0.0521 (13)	0.0747 (15)	0.0006 (9)	−0.0069 (10)	0.0069 (11)
C8	0.0377 (12)	0.0649 (16)	0.109 (2)	−0.0009 (11)	−0.0211 (13)	0.0024 (15)
C9	0.0599 (15)	0.0682 (17)	0.092 (2)	0.0121 (13)	−0.0366 (14)	−0.0202 (14)
C10	0.0685 (16)	0.0701 (17)	0.0666 (15)	0.0199 (13)	−0.0167 (12)	−0.0181 (12)
C11	0.0458 (11)	0.0591 (14)	0.0669 (14)	0.0107 (10)	−0.0083 (10)	−0.0125 (11)
C12	0.0297 (8)	0.0354 (9)	0.0428 (9)	−0.0003 (7)	−0.0044 (7)	−0.0007 (7)
C13	0.0375 (9)	0.0446 (11)	0.0435 (10)	−0.0029 (8)	−0.0051 (7)	0.0024 (8)
C14	0.0498 (12)	0.0439 (11)	0.0592 (12)	0.0003 (9)	−0.0152 (10)	0.0090 (9)
C15	0.0416 (11)	0.0439 (12)	0.0766 (15)	0.0103 (9)	−0.0150 (10)	−0.0058 (10)
C16	0.0446 (11)	0.0635 (14)	0.0643 (14)	0.0154 (11)	0.0036 (10)	−0.0086 (11)
C17	0.0438 (11)	0.0553 (12)	0.0474 (11)	0.0104 (9)	0.0026 (8)	0.0054 (9)
C18	0.0450 (11)	0.0517 (12)	0.0556 (12)	−0.0056 (9)	−0.0084 (9)	−0.0063 (10)
C19	0.082 (2)	0.078 (2)	0.119 (3)	−0.0299 (17)	−0.0414 (19)	0.0050 (19)

### 3-(2-Chloroethyl)-r-2,c-6-diphenylpiperidin-4-one (I) Geometric parameters (Å, º)

**Table:**

Cl1—C19	1.801 (4)	C8—C9	1.363 (4)
O1—C3	1.209 (3)	C9—H9	0.9300
N1—H1	0.86 (2)	C9—C10	1.379 (4)
N1—C1	1.469 (2)	C10—H10	0.9300
N1—C5	1.466 (2)	C10—C11	1.379 (3)
C1—H1A	0.9800	C11—H11	0.9300
C1—C2	1.551 (2)	C12—C13	1.382 (3)
C1—C12	1.511 (2)	C12—C17	1.388 (3)
C2—H2	0.9800	C13—H13	0.9300
C2—C3	1.517 (3)	C13—C14	1.391 (3)
C2—C18	1.528 (3)	C14—H14	0.9300
C3—C4	1.502 (3)	C14—C15	1.367 (3)
C4—H4A	0.9700	C15—H15	0.9300
C4—H4B	0.9700	C15—C16	1.376 (3)
C4—C5	1.537 (3)	C16—H16	0.9300
C5—H5	0.9800	C16—C17	1.383 (3)
C5—C6	1.512 (3)	C17—H17	0.9300
C6—C7	1.386 (3)	C18—H18A	0.9700
C6—C11	1.389 (3)	C18—H18B	0.9700
C7—H7	0.9300	C18—C19	1.490 (4)
C7—C8	1.395 (4)	C19—H19A	0.9700
C8—H8	0.9300	C19—H19B	0.9700
			
C1—N1—H1	112.3 (15)	C8—C9—H9	120.1
C5—N1—H1	109.1 (16)	C8—C9—C10	119.8 (2)
C5—N1—C1	111.13 (15)	C10—C9—H9	120.1
N1—C1—H1A	108.8	C9—C10—H10	120.0
N1—C1—C2	108.14 (15)	C9—C10—C11	120.1 (3)
N1—C1—C12	110.41 (15)	C11—C10—H10	120.0
C2—C1—H1A	108.8	C6—C11—H11	119.5
C12—C1—H1A	108.8	C10—C11—C6	121.0 (2)
C12—C1—C2	111.71 (15)	C10—C11—H11	119.5
C1—C2—H2	106.9	C13—C12—C1	120.73 (17)
C3—C2—C1	110.20 (15)	C13—C12—C17	118.22 (18)
C3—C2—H2	106.9	C17—C12—C1	121.04 (17)
C3—C2—C18	112.34 (17)	C12—C13—H13	119.7
C18—C2—C1	113.15 (17)	C12—C13—C14	120.66 (19)
C18—C2—H2	106.9	C14—C13—H13	119.7
O1—C3—C2	122.9 (2)	C13—C14—H14	119.8
O1—C3—C4	120.7 (2)	C15—C14—C13	120.5 (2)
C4—C3—C2	116.46 (17)	C15—C14—H14	119.8
C3—C4—H4A	109.2	C14—C15—H15	120.3
C3—C4—H4B	109.2	C14—C15—C16	119.5 (2)
C3—C4—C5	111.87 (17)	C16—C15—H15	120.3
H4A—C4—H4B	107.9	C15—C16—H16	119.8
C5—C4—H4A	109.2	C15—C16—C17	120.4 (2)
C5—C4—H4B	109.2	C17—C16—H16	119.8
N1—C5—C4	107.14 (16)	C12—C17—H17	119.6
N1—C5—H5	108.9	C16—C17—C12	120.8 (2)
N1—C5—C6	111.71 (16)	C16—C17—H17	119.6
C4—C5—H5	108.9	C2—C18—H18A	108.5
C6—C5—C4	111.33 (17)	C2—C18—H18B	108.5
C6—C5—H5	108.9	H18A—C18—H18B	107.5
C7—C6—C5	120.0 (2)	C19—C18—C2	115.0 (2)
C7—C6—C11	118.5 (2)	C19—C18—H18A	108.5
C11—C6—C5	121.46 (19)	C19—C18—H18B	108.5
C6—C7—H7	120.0	Cl1—C19—H19A	109.6
C6—C7—C8	120.1 (3)	Cl1—C19—H19B	109.6
C8—C7—H7	120.0	C18—C19—Cl1	110.3 (2)
C7—C8—H8	119.7	C18—C19—H19A	109.6
C9—C8—C7	120.6 (2)	C18—C19—H19B	109.6
C9—C8—H8	119.7	H19A—C19—H19B	108.1
			
O1—C3—C4—C5	136.1 (2)	C4—C5—C6—C11	−65.2 (3)
N1—C1—C2—C3	−52.8 (2)	C5—N1—C1—C2	68.10 (18)
N1—C1—C2—C18	−179.46 (16)	C5—N1—C1—C12	−169.41 (15)
N1—C1—C12—C13	123.97 (18)	C5—C6—C7—C8	−177.2 (2)
N1—C1—C12—C17	−57.6 (2)	C5—C6—C11—C10	177.0 (2)
N1—C5—C6—C7	−128.3 (2)	C6—C7—C8—C9	0.1 (4)
N1—C5—C6—C11	54.5 (3)	C7—C6—C11—C10	−0.2 (4)
C1—N1—C5—C4	−67.8 (2)	C7—C8—C9—C10	−0.2 (4)
C1—N1—C5—C6	170.05 (16)	C8—C9—C10—C11	0.0 (4)
C1—C2—C3—O1	−137.1 (2)	C9—C10—C11—C6	0.2 (4)
C1—C2—C3—C4	43.6 (2)	C11—C6—C7—C8	0.1 (3)
C1—C2—C18—C19	−158.8 (2)	C12—C1—C2—C3	−174.45 (16)
C1—C12—C13—C14	177.64 (18)	C12—C1—C2—C18	58.8 (2)
C1—C12—C17—C16	−178.0 (2)	C12—C13—C14—C15	0.4 (3)
C2—C1—C12—C13	−115.65 (19)	C13—C12—C17—C16	0.5 (3)
C2—C1—C12—C17	62.8 (2)	C13—C14—C15—C16	0.4 (3)
C2—C3—C4—C5	−44.6 (3)	C14—C15—C16—C17	−0.8 (4)
C2—C18—C19—Cl1	64.5 (3)	C15—C16—C17—C12	0.3 (4)
C3—C2—C18—C19	75.6 (3)	C17—C12—C13—C14	−0.9 (3)
C3—C4—C5—N1	53.6 (2)	C18—C2—C3—O1	−9.9 (3)
C3—C4—C5—C6	176.03 (18)	C18—C2—C3—C4	170.81 (19)
C4—C5—C6—C7	112.0 (2)		

### 3-(2-Chloroethyl)-r-2,c-6-diphenylpiperidin-4-one (I) Hydrogen-bond geometry (Å, º)

Cg3 is the centroid of the C12–C17 ring.

**Table:**

*D*—H···*A*	*D*—H	H···*A*	*D*···*A*	*D*—H···*A*
N1—H1···O1^i^	0.86 (2)	2.52 (2)	3.335 (2)	158 (2)
C4—H4*A*···*Cg*3^ii^	0.97	2.79	3.665 (2)	150

Symmetry codes: (i) −*x*+1, *y*+1/2, −*z*+1/2; (ii) −*x*+1, *y*−1/2, −*z*+1/2.

### 3-(2-chloroethyl)-r-2,c-6-bis(4-fluorophenyl)piperidin-4-one (II) Crystal data

**Table:**

C_19_H_18_ClF_2_NO	*F*(000) = 728
*M**_r_* = 349.79	*D*_x_ = 1.354 Mg m^−^^3^
Monoclinic, *P*2_1_/*c*	Cu *K*α radiation, λ = 1.54184 Å
*a* = 5.5105 (2) Å	Cell parameters from 2364 reflections
*b* = 24.2612 (6) Å	θ = 3.4–71.3°
*c* = 12.8622 (3) Å	µ = 2.20 mm^−^^1^
β = 93.809 (3)°	*T* = 293 K
*V* = 1715.77 (9) Å^3^	Prism, colourless
*Z* = 4	0.34 × 0.16 × 0.14 mm

### 3-(2-chloroethyl)-r-2,c-6-bis(4-fluorophenyl)piperidin-4-one (II) Data collection

**Table:**

Rigaku Oxford Diffraction diffractometer	3267 independent reflections
Radiation source: fine-focus sealed X-ray tube, Enhance (Cu) X-ray Source	2702 reflections with *I* > 2σ(*I*)
Graphite monochromator	*R*_int_ = 0.020
Detector resolution: 16.0416 pixels mm^-1^	θ_max_ = 71.3°, θ_min_ = 3.6°
ω scans	*h* = −6→6
Absorption correction: multi-scan (CrysAlis PRO; Rigaku OD, 2015)	*k* = −29→16
*T*_min_ = 0.524, *T*_max_ = 1.000	*l* = −14→15
6548 measured reflections	

### 3-(2-chloroethyl)-r-2,c-6-bis(4-fluorophenyl)piperidin-4-one (II) Refinement

**Table:**

Refinement on *F*^2^	Hydrogen site location: mixed
Least-squares matrix: full	H atoms treated by a mixture of independent and constrained refinement
*R*[*F*^2^ > 2σ(*F*^2^)] = 0.046	*w* = 1/[σ^2^(*F*_o_^2^) + (0.0592*P*)^2^ + 0.5453*P*] where *P* = (*F*_o_^2^ + 2*F*_c_^2^)/3
*wR*(*F*^2^) = 0.131	(Δ/σ)_max_ < 0.001
*S* = 1.04	Δρ_max_ = 0.34 e Å^−^^3^
3267 reflections	Δρ_min_ = −0.38 e Å^−^^3^
222 parameters	Extinction correction: SHELXL2018 (Sheldrick, 2015b), Fc^*^=kFc[1+0.001xFc^2^λ^3^/sin(2θ)]^-1/4^
0 restraints	Extinction coefficient: 0.0058 (5)
Primary atom site location: dual	

### 3-(2-chloroethyl)-r-2,c-6-bis(4-fluorophenyl)piperidin-4-one (II) Special details

**Table:**

Geometry. All esds (except the esd in the dihedral angle between two l.s. planes) are estimated using the full covariance matrix. The cell esds are taken into account individually in the estimation of esds in distances, angles and torsion angles; correlations between esds in cell parameters are only used when they are defined by crystal symmetry. An approximate (isotropic) treatment of cell esds is used for estimating esds involving l.s. planes.

### 3-(2-chloroethyl)-r-2,c-6-bis(4-fluorophenyl)piperidin-4-one (II) Fractional atomic coordinates and isotropic or equivalent isotropic displacement parameters (Å^2^)

**Table:**

	*x*	*y*	*z*	*U*_iso_*/*U*_eq_	
Cl1	0.15647 (18)	0.46455 (3)	0.38176 (8)	0.1057 (3)	
F1	−0.2069 (3)	0.04268 (6)	0.63058 (13)	0.0854 (5)	
F2	0.6123 (3)	0.45967 (6)	0.88365 (11)	0.0747 (4)	
O1	0.4499 (4)	0.29713 (7)	0.28005 (11)	0.0702 (5)	
N1	0.3190 (3)	0.27067 (6)	0.57146 (11)	0.0396 (4)	
H1	0.366 (4)	0.2579 (9)	0.6346 (17)	0.043 (5)*	
C1	0.3477 (4)	0.29649 (8)	0.36027 (14)	0.0489 (5)	
C2	0.1985 (4)	0.24790 (9)	0.39147 (14)	0.0528 (5)	
H2A	0.199144	0.219410	0.338582	0.063*	
H2B	0.031521	0.259163	0.398695	0.063*	
C3	0.3112 (4)	0.22560 (7)	0.49651 (14)	0.0414 (4)	
H3	0.478176	0.213743	0.486823	0.050*	
C4	0.4808 (3)	0.31538 (7)	0.54325 (13)	0.0384 (4)	
H4	0.639887	0.299519	0.530846	0.046*	
C5	0.3752 (4)	0.34225 (7)	0.44054 (13)	0.0431 (4)	
H5	0.212671	0.356346	0.452333	0.052*	
C6	0.5288 (4)	0.39034 (8)	0.40516 (16)	0.0530 (5)	
H6A	0.582661	0.412091	0.465641	0.064*	
H6B	0.672413	0.375749	0.375148	0.064*	
C7	0.3962 (5)	0.42751 (11)	0.3263 (2)	0.0737 (7)	
H7A	0.329082	0.405487	0.268405	0.088*	
H7B	0.510404	0.453477	0.299551	0.088*	
C8	0.1707 (3)	0.17694 (7)	0.53451 (13)	0.0389 (4)	
C9	0.2447 (4)	0.12399 (8)	0.51246 (15)	0.0462 (4)	
H9	0.382523	0.118931	0.475566	0.055*	
C10	0.1180 (4)	0.07832 (8)	0.54414 (17)	0.0548 (5)	
H10	0.166749	0.042824	0.527870	0.066*	
C11	−0.0797 (4)	0.08699 (8)	0.59972 (16)	0.0534 (5)	
C12	−0.1588 (4)	0.13849 (9)	0.62493 (17)	0.0552 (5)	
H12	−0.294820	0.142967	0.663223	0.066*	
C13	−0.0313 (4)	0.18376 (8)	0.59200 (16)	0.0480 (5)	
H13	−0.081684	0.219075	0.608620	0.058*	
C14	0.5130 (3)	0.35521 (7)	0.63374 (13)	0.0378 (4)	
C15	0.3388 (4)	0.39397 (8)	0.65460 (15)	0.0488 (5)	
H15	0.197012	0.396256	0.611350	0.059*	
C16	0.3714 (4)	0.42936 (8)	0.73856 (16)	0.0534 (5)	
H16	0.254041	0.455500	0.751883	0.064*	
C17	0.5796 (4)	0.42507 (8)	0.80138 (15)	0.0513 (5)	
C18	0.7540 (4)	0.38685 (10)	0.78520 (18)	0.0628 (6)	
H18	0.892640	0.384155	0.830246	0.075*	
C19	0.7201 (4)	0.35205 (9)	0.70008 (17)	0.0544 (5)	
H19	0.838816	0.326106	0.687407	0.065*	

### 3-(2-chloroethyl)-r-2,c-6-bis(4-fluorophenyl)piperidin-4-one (II) Atomic displacement parameters (Å^2^)

**Table:**

	*U*^11^	*U*^22^	*U*^33^	*U*^12^	*U*^13^	*U*^23^
Cl1	0.1146 (7)	0.0738 (5)	0.1305 (7)	0.0231 (4)	0.0211 (5)	0.0356 (5)
F1	0.1086 (12)	0.0563 (8)	0.0919 (11)	−0.0353 (8)	0.0103 (9)	0.0197 (7)
F2	0.1015 (11)	0.0585 (8)	0.0632 (8)	−0.0144 (7)	−0.0006 (7)	−0.0275 (6)
O1	0.1093 (14)	0.0624 (9)	0.0408 (8)	−0.0254 (9)	0.0187 (8)	−0.0040 (7)
N1	0.0553 (9)	0.0310 (7)	0.0325 (7)	−0.0058 (6)	0.0018 (6)	0.0019 (6)
C1	0.0664 (12)	0.0454 (10)	0.0342 (9)	−0.0112 (9)	−0.0015 (8)	0.0048 (7)
C2	0.0737 (13)	0.0469 (10)	0.0371 (9)	−0.0191 (10)	−0.0017 (9)	−0.0026 (8)
C3	0.0506 (10)	0.0337 (8)	0.0401 (9)	−0.0062 (7)	0.0046 (7)	−0.0015 (7)
C4	0.0453 (9)	0.0328 (8)	0.0370 (8)	−0.0033 (7)	0.0019 (7)	−0.0006 (7)
C5	0.0549 (10)	0.0373 (9)	0.0369 (9)	−0.0093 (8)	0.0023 (7)	0.0048 (7)
C6	0.0693 (13)	0.0430 (10)	0.0472 (10)	−0.0153 (9)	0.0075 (9)	0.0045 (8)
C7	0.101 (2)	0.0561 (13)	0.0645 (14)	−0.0090 (13)	0.0123 (13)	0.0215 (11)
C8	0.0465 (9)	0.0325 (8)	0.0372 (8)	−0.0037 (7)	−0.0008 (7)	−0.0006 (6)
C9	0.0523 (10)	0.0370 (9)	0.0494 (10)	−0.0009 (8)	0.0038 (8)	−0.0060 (8)
C10	0.0726 (14)	0.0295 (9)	0.0611 (12)	−0.0003 (9)	−0.0048 (10)	−0.0018 (8)
C11	0.0662 (12)	0.0415 (10)	0.0514 (11)	−0.0176 (9)	−0.0049 (9)	0.0103 (8)
C12	0.0557 (12)	0.0545 (12)	0.0563 (11)	−0.0090 (9)	0.0105 (9)	0.0017 (9)
C13	0.0539 (11)	0.0356 (9)	0.0552 (11)	0.0002 (8)	0.0083 (9)	−0.0019 (8)
C14	0.0471 (9)	0.0305 (8)	0.0356 (8)	−0.0062 (7)	0.0024 (7)	0.0009 (6)
C15	0.0505 (10)	0.0478 (10)	0.0475 (10)	0.0012 (8)	−0.0016 (8)	−0.0052 (8)
C16	0.0641 (12)	0.0417 (10)	0.0550 (11)	0.0037 (9)	0.0085 (9)	−0.0073 (8)
C17	0.0715 (13)	0.0382 (9)	0.0443 (10)	−0.0151 (9)	0.0043 (9)	−0.0097 (8)
C18	0.0622 (13)	0.0659 (14)	0.0577 (12)	−0.0028 (11)	−0.0159 (10)	−0.0153 (11)
C19	0.0557 (11)	0.0492 (11)	0.0566 (11)	0.0074 (9)	−0.0079 (9)	−0.0108 (9)

### 3-(2-chloroethyl)-r-2,c-6-bis(4-fluorophenyl)piperidin-4-one (II) Geometric parameters (Å, º)

**Table:**

Cl1—C7	1.785 (3)	C7—H7A	0.9700
F1—C11	1.357 (2)	C7—H7B	0.9700
F2—C17	1.353 (2)	C8—C9	1.383 (3)
O1—C1	1.208 (2)	C8—C13	1.386 (3)
N1—H1	0.89 (2)	C9—H9	0.9300
N1—C3	1.457 (2)	C9—C10	1.385 (3)
N1—C4	1.465 (2)	C10—H10	0.9300
C1—C2	1.507 (3)	C10—C11	1.359 (3)
C1—C5	1.517 (3)	C11—C12	1.369 (3)
C2—H2A	0.9700	C12—H12	0.9300
C2—H2B	0.9700	C12—C13	1.385 (3)
C2—C3	1.547 (3)	C13—H13	0.9300
C3—H3	0.9800	C14—C15	1.383 (3)
C3—C8	1.511 (2)	C14—C19	1.381 (3)
C4—H4	0.9800	C15—H15	0.9300
C4—C5	1.551 (2)	C15—C16	1.382 (3)
C4—C14	1.514 (2)	C16—H16	0.9300
C5—H5	0.9800	C16—C17	1.363 (3)
C5—C6	1.528 (2)	C17—C18	1.361 (3)
C6—H6A	0.9700	C18—H18	0.9300
C6—H6B	0.9700	C18—C19	1.385 (3)
C6—C7	1.509 (3)	C19—H19	0.9300
			
C3—N1—H1	109.6 (14)	C6—C7—H7A	109.3
C3—N1—C4	112.57 (14)	C6—C7—H7B	109.3
C4—N1—H1	109.7 (14)	H7A—C7—H7B	107.9
O1—C1—C2	122.08 (19)	C9—C8—C3	119.67 (17)
O1—C1—C5	122.75 (18)	C9—C8—C13	118.58 (17)
C2—C1—C5	115.02 (16)	C13—C8—C3	121.75 (16)
C1—C2—H2A	110.1	C8—C9—H9	119.3
C1—C2—H2B	110.1	C8—C9—C10	121.42 (18)
C1—C2—C3	108.16 (16)	C10—C9—H9	119.3
H2A—C2—H2B	108.4	C9—C10—H10	121.0
C3—C2—H2A	110.1	C11—C10—C9	117.93 (18)
C3—C2—H2B	110.1	C11—C10—H10	121.0
N1—C3—C2	107.94 (15)	F1—C11—C10	118.6 (2)
N1—C3—H3	108.5	F1—C11—C12	118.4 (2)
N1—C3—C8	111.46 (14)	C10—C11—C12	123.02 (19)
C2—C3—H3	108.5	C11—C12—H12	120.8
C8—C3—C2	111.80 (15)	C11—C12—C13	118.36 (19)
C8—C3—H3	108.5	C13—C12—H12	120.8
N1—C4—H4	108.4	C8—C13—H13	119.7
N1—C4—C5	108.78 (14)	C12—C13—C8	120.67 (18)
N1—C4—C14	108.93 (13)	C12—C13—H13	119.7
C5—C4—H4	108.4	C15—C14—C4	122.38 (16)
C14—C4—H4	108.4	C19—C14—C4	119.30 (17)
C14—C4—C5	113.86 (14)	C19—C14—C15	118.29 (17)
C1—C5—C4	106.67 (15)	C14—C15—H15	119.4
C1—C5—H5	108.0	C16—C15—C14	121.19 (19)
C1—C5—C6	112.85 (16)	C16—C15—H15	119.4
C4—C5—H5	108.0	C15—C16—H16	120.8
C6—C5—C4	112.99 (16)	C17—C16—C15	118.48 (19)
C6—C5—H5	108.0	C17—C16—H16	120.8
C5—C6—H6A	108.8	F2—C17—C16	118.6 (2)
C5—C6—H6B	108.8	F2—C17—C18	118.92 (19)
H6A—C6—H6B	107.7	C18—C17—C16	122.44 (18)
C7—C6—C5	113.76 (19)	C17—C18—H18	120.8
C7—C6—H6A	108.8	C17—C18—C19	118.5 (2)
C7—C6—H6B	108.8	C19—C18—H18	120.8
Cl1—C7—H7A	109.3	C14—C19—C18	121.1 (2)
Cl1—C7—H7B	109.3	C14—C19—H19	119.4
C6—C7—Cl1	111.78 (17)	C18—C19—H19	119.4
			
F1—C11—C12—C13	−178.99 (19)	C4—C5—C6—C7	−162.48 (18)
F2—C17—C18—C19	−179.3 (2)	C4—C14—C15—C16	179.14 (18)
O1—C1—C2—C3	120.0 (2)	C4—C14—C19—C18	−178.5 (2)
O1—C1—C5—C4	−120.4 (2)	C5—C1—C2—C3	−55.6 (2)
O1—C1—C5—C6	4.2 (3)	C5—C4—C14—C15	43.2 (2)
N1—C3—C8—C9	−144.20 (17)	C5—C4—C14—C19	−138.73 (19)
N1—C3—C8—C13	35.4 (2)	C5—C6—C7—Cl1	67.6 (2)
N1—C4—C5—C1	−56.49 (19)	C8—C9—C10—C11	−1.3 (3)
N1—C4—C5—C6	178.94 (15)	C9—C8—C13—C12	−1.2 (3)
N1—C4—C14—C15	−78.4 (2)	C9—C10—C11—F1	179.48 (19)
N1—C4—C14—C19	99.7 (2)	C9—C10—C11—C12	0.4 (3)
C1—C2—C3—N1	56.4 (2)	C10—C11—C12—C13	0.1 (3)
C1—C2—C3—C8	179.37 (16)	C11—C12—C13—C8	0.3 (3)
C1—C5—C6—C7	76.4 (2)	C13—C8—C9—C10	1.7 (3)
C2—C1—C5—C4	55.1 (2)	C14—C4—C5—C1	−178.17 (15)
C2—C1—C5—C6	179.78 (18)	C14—C4—C5—C6	57.3 (2)
C2—C3—C8—C9	94.9 (2)	C14—C15—C16—C17	−0.4 (3)
C2—C3—C8—C13	−85.5 (2)	C15—C14—C19—C18	−0.3 (3)
C3—N1—C4—C5	65.35 (19)	C15—C16—C17—F2	179.98 (18)
C3—N1—C4—C14	−170.01 (15)	C15—C16—C17—C18	−0.9 (3)
C3—C8—C9—C10	−178.71 (18)	C16—C17—C18—C19	1.6 (4)
C3—C8—C13—C12	179.24 (18)	C17—C18—C19—C14	−1.0 (4)
C4—N1—C3—C2	−64.5 (2)	C19—C14—C15—C16	1.0 (3)
C4—N1—C3—C8	172.34 (15)		

### 3-(2-chloroethyl)-r-2,c-6-bis(4-fluorophenyl)piperidin-4-one (II) Hydrogen-bond geometry (Å, º)

**Table:**

*D*—H···*A*	*D*—H	H···*A*	*D*···*A*	*D*—H···*A*
N1—H1···O1^i^	0.89 (2)	2.32 (2)	3.189 (2)	165 (2)
C9—H9···F2^ii^	0.93	2.61	3.378 (2)	140
C10—H10···F2^iii^	0.93	2.58	3.343 (2)	139
C12—H12···O1^iv^	0.93	2.57	3.412 (3)	150
C16—H16···F1^v^	0.93	2.62	3.379 (2)	139

Symmetry codes: (i) *x*, −*y*+1/2, *z*+1/2; (ii) *x*, −*y*+1/2, *z*−1/2; (iii) −*x*+1, *y*−1/2, −*z*+3/2; (iv) *x*−1, −*y*+1/2, *z*+1/2; (v) −*x*, *y*+1/2, −*z*+3/2.
